# Synergies revealed: RNA-seq study of *C. acetobutylicum* and *C. carboxidivorans* co-cultured in the presence of conductive materials

**DOI:** 10.1128/spectrum.03262-25

**Published:** 2026-03-02

**Authors:** L. Feliu-Paradeda, Q. Amorós-Espuña, E. Perona-Vico, E. Casals, A. Esteve-Codina, S. Puig, L. Bañeras

**Affiliations:** 1 Molecular Microbial Ecology Group, Institute of Aquatic Ecology, University of Girona 16738, Girona, Spain; 2 Centro Nacional de Análisis Genómico (CNAG), Barcelona, Spain; 3 Universitat de Barcelona (UB) 16724, Barcelona, Spain; 4 LEQUiA, Institute of the Environment, University of Girona, Girona, Spain; University of Minnesota Twin Cities, St. Paul, Minnesota, USA

**Keywords:** *Clostridium*, co-culture dynamics, gene expression patterns, RNA sequencing, bacterial cooperation

## Abstract

Co-cultures can improve substrate utilization and product yields, yet the dynamics between species remain highly variable and poorly understood. In this study, we investigated the metabolic and transcriptional interplay between *Clostridium acetobutylicum* and *Clostridium carboxidivorans* when co-cultured in the presence of activated carbon or magnetite, with the aim of evaluating these materials as metabolism enhancers. Fermentation profiling showed that magnetite accelerated glucose consumption and favored acid over alcohol production, with butyrate and acetate reaching yields of 0.65 and 0.62 mol/mol glucose, respectively. Alcohols (ethanol and butanol) accumulated in late fermentation and occurred concomitantly to a metabolic shift from acidogenesis to solventogenesis, potentially driven by interspecies dynamics as RNA-seq data suggested. RNA-seq analysis detected 7,369 genes and revealed *C. carboxidivorans* dominated in early fermentation, and *C. acetobutylicum* was activated later (in view of the number of reads detected for each species). Magnetite-treated samples displayed the most transcriptional variation, and species-specific patterns emerged. Changes in electron-active genes (e.g., *hydA* and *rnf* genes) suggest enhanced redox communication, highlighting the capacity of conductive materials to influence metabolic flow and regulatory pathways in microbial consortia, opening possibilities for improved bioproduction and carbon utilization.

**IMPORTANCE:**

Microbial co-cultures offer a promising strategy to expand metabolic capabilities beyond those of individual strains, yet their internal coordination remains poorly understood. This study demonstrates that conductive materials not only accelerate substrate utilization but also modulate cooperation in a co-culture of *Clostridium carboxidivorans* and *Clostridium acetobutylicum*. According to gene expression levels, we demonstrate a clear temporal division of labor between the two partners, with *C. carboxidivorans* initiating acidogenesis and *C. acetobutylicum* later driving solventogenesis. Magnetite and activated carbon addition had little effect, but changes in the expression pattern of electron-active genes (*hydA* and *rnf*) could be detected for the two species. Understanding and controlling these dynamics are key to optimizing co-cultures for industrial fermentation and biofuel production.

## OBSERVATION

The co-culture of *Clostridium* strains offers significant advantages in fermentation processes (i.e., enhancing substrate utilization and improving product yield and spectrum) (1). In particular, controlling the growth and activity of individual strains is essential for successful co-culturing and can prevent one species from outcompeting the other (2). In a previous work, we observed that the addition of the semiconductor-like magnetite particles to a co-culture of *C. carboxidivorans* (acetogenic) and *C. acetobutylicum* (solventogenic) improved the production of acids and alcohols, especially during the onset of the stationary phase (3). Since no improvement was detected in either the co-culture without the addition of magnetite or the pure cultures, we hypothesized magnetite served as an electric capacitor by either absorbing reducing equivalents or donating electrons to *Clostridium*, thus partially controlling the physiology of the two partners. This similar reaction was observed by Byrne and colleagues in *Geobacter sulfurreducens* and *Rhodopseudomonas palustris* TIE-1 co-cultures (4). Ideally, physiological changes should be directed by changes in the gene expression patterns (5), which are assessed in this short communication.

In order to detect changes in the gene expression directed by the presence of weakly conductive activated carbon (AC) or semiconductor-like magnetite (Fe_3_O_4_, MAG) particles, co-cultures of *C. carboxidivorans* and *C. acetobutylicum* were set, and acid and alcohol production, growth kinetics, and the gene expression dynamics of both species were monitored. The fermentation experiments consisted of four sacrificial bottles (for each treatment) incubated at 37°C at 130 rpm, with 150 mL of a synthetic medium at an initial glucose concentration set at 20 g/L. Medium composition, co-culture inoculation, magnetite or activated carbon supplementation, and RNA extraction were performed as reported in previous works (3, 6).

**Fig 1:**
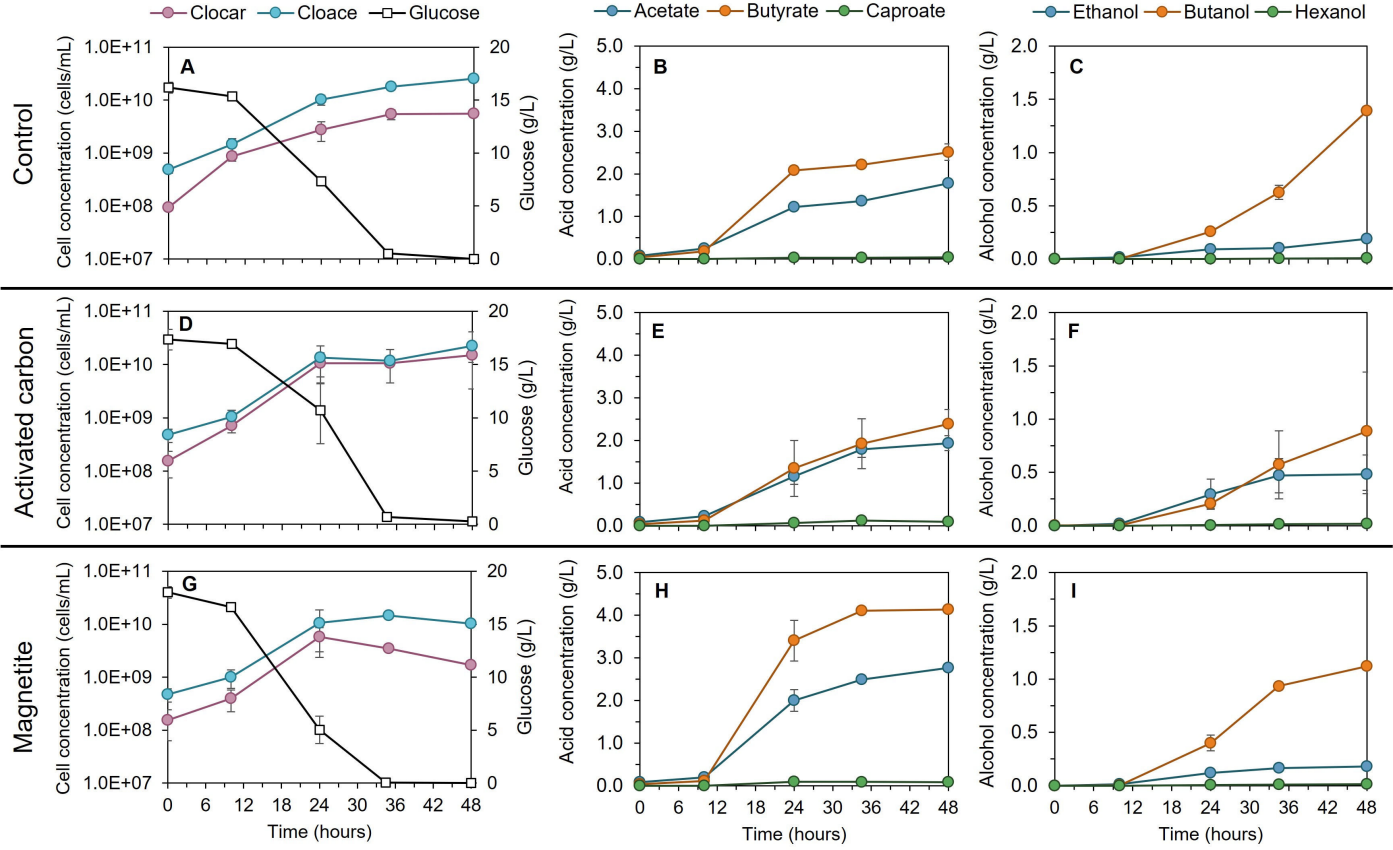
Glucose consumption and cell growth (**A, D, G**), acid (**B, E, H**), and alcohol (**C, F, I**) production of *C. acetobutylicum/C. carboxidivorans* co-culture under control, activated carbon, or magnetite supplementation experiments. Error bars represent the variability of the replicates at each time point. Clocar: *C. carboxidivorans*; Cloace: *C. acetobutylicum*.

Growth and alcohol production in the three treatments (control, AC, and MAG) occurred as expected. Similar cell densities were achieved, regardless of the treatment (3.2·10^10^ and 3.7·10^10^ cells/mL, respectively), but were slightly lower for *C. carboxidivorans* in MAG (Fig. 1). Glucose consumption in MAG (0.534 ± 0.051 g/L·h^−1^) occurred faster during the first 24 h compared to control (0.325 ± 0.028 g/L·h^−1^, *P*-value < 0.01). In fact, a positive effect of the addition of redox active compounds (magnetite or ferrous iron) on sugar consumption rate has been previously observed in pure cultures of *Clostridium butyricum* (7) and *C. beijerinckii* (8) or in mixed cultures (3), revealing an active role of iron in *Clostridium* metabolism. Magnetite promoted the production of acids (83.9% of acid selectivity), reaching the highest acid production yields (0.65 and 0.62 mol/mol glucose for butyrate and acetate, respectively). At the end of the fermentation, the lowest acid-to-alcohol ratio was found in control (2.71 ± 0.14 g/g).

**Fig 2:**
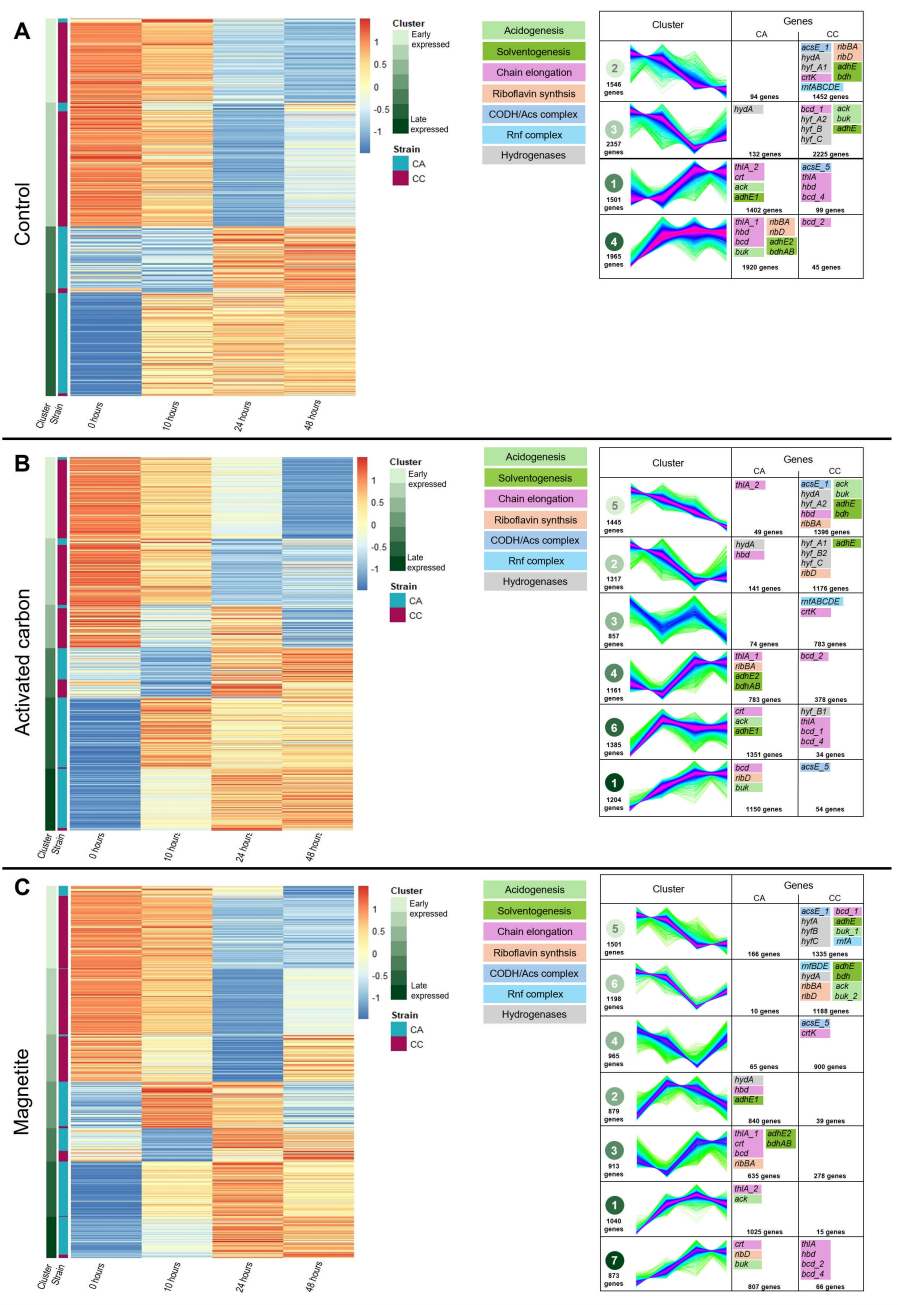
Heatmap showing the Z-score obtained with *mfuzz* gene clustering analysis and the different clusters obtained for control (**A**), activated carbon (**B**), and magnetite (**C**) supplementations. In the heatmap, the first column indicates the cluster expression (green), and the second column indicates the strain (*C. acetobutylicum* in blue; *C. carboxidivorans* in pink). The Mfuzz Z-score values are represented in a color scale from blue to red. The number of genes assigned to each Mfuzz cluster is indicated in the table.

Maximum acid and alcohol production rates occurred sequentially in all treatments. Both strains used are alcohologenic strains and showed a parallel growth response in the experiment. However, it was not clear if a dynamic alternation of oxidative (production of acids) to reductive (alcohol production) reactions could be differentiated for the two strains. To investigate this further, an RNA-seq analysis was performed. RNA-seq libraries were prepared with the Illumina Stranded Total RNA Prep with Ribo-Zero Plus Microbiome and sequenced on NovaSeq 6000 (Illumina) with a read length of 2 × 51 bp. RNA-seq reads were mapped against the genomes of *Clostridium acetobutylicum* ATCC 824 (gca_000008765) and *Clostridium carboxidivorans* P7 (gca_000175595) with STAR 2.7.8a using ENCODE parameters (9). Genes were quantified with RSEM 1.3.0 (10). Soft clustering, a method specifically designed to identify genes with similar temporal expression profiles in time-course experiments, was performed using the R package Mfuzz (11). The genome annotations of *Clostridium acetobutylicum* and *Clostridium carboxidivorans* comprise 4,001 and 5,447 genes, respectively. Approximately, 2.6 × 10^8^ and 0.7 × 10^8^ reads were mapped to *C. acetobutylicum* and *C. carboxidivorans*, respectively, being relatively larger for *C. carboxidivorans* at the beginning compared to the end of the fermentation. After filtering out genes that were not expressed in any of the analyzed samples, the resulting data sets included 3,548 genes for *C. acetobutylicum* and 3,821 genes for *C. carboxidivorans*, yielding a combined total of 7,369 expressed genes used for downstream analyses. The initial number of clusters (*K*) for Mfuzz soft clustering was set to 12. After each run, *K* was iteratively adjusted by evaluating both the Mfuzz overlap diagram and GO term enrichment results. When clusters showed high membership overlap and shared a substantial number of enriched GO terms (compared to other cluster pairs), *K* was reduced to minimize redundancy. This process was repeated until a minimal overlap and functional distinction between clusters were achieved. Four Mfuzz expression clusters were identified in control, six in the activated carbon, and seven in the magnetite treatment. For an easier comparison among treatments, Mfuzz clusters were organized according to an earlier or late expression dynamics (Fig. 2).

RNA-seq results confirmed the metabolic alternation of the two strains. The Mfuzz scores (membership value) represent the degree to which a gene conforms to the expression pattern of a given cluster, ranging from 0 to 1. Unlike expression metrics, such as RPKM, which quantify expression, the Mfuzz score reflects similarity in expression dynamics across conditions or time points after normalization and standardization of the data. In this sense, *C. carboxidivorans*-related genes mainly clustered to clusters 2 and 3 (control), to clusters 5, 2 and 3 (activated carbon), or to 5, 6, and 4 (magnetite), that showed a higher activity at the start of the fermentation (<10 h). In contrast, *C. acetobutylicum*-related genes mainly clustered in Mfuzz clusters showing an increasing dynamics as fermentation progresses. Since both species grew concomitantly during the 48 h of fermentation, RNA-seq results revealed a sequential metabolic interaction between the two partners. *C. carboxidivorans* initially engaged in acidogenesis while disposing reducing equivalents, and *C. acetobutylicum* followed with alcohol production by the upregulation of solventogenesis genes during the later stages of fermentation.

The addition of electron-active compounds (activated carbon or magnetite) was expected to improve electron transfer events between the two *Clostridium*. In order to assess this, we analyzed the expression of genes related to riboflavin synthesis, synthesized by the *ribGBAH* operon present in both *Clostridium* species (12, 13), and ferredoxin redox cycling systems, such as hydrogenases, and Bcd and Rnf complexes. All these proteins are thought to facilitate interspecies electron transfer by acting as free-form electron shuttles (14, 15) or promote the cycling of the NADH/NAD^+^ equilibrium (16). The majority of *rib* genes remained in the same Mfuzz cluster in all three growing conditions and were considered to have negligible activity in electron transfer. Conversely, *rnfABCDE* genes from *C. carboxidivorans* changed from a repression pattern in control and magnetite to a transiently expressed pattern in activated carbon. It should be noted that the Rnf complex is exclusively found in *C. carboxidivorans* (17). Similar results were obtained with hydrogenase genes (*hydA*) of *C. acetobutylicum* with magnetite supplementation. The Rnf complex has been identified as potential entry points for extracellular electrons in the metabolism of *C. autoethanogenum* and *C. ljungdahlii* (18, 19). Thus, their changes in expression compared to control could point to an involvement in electron transfer between the two bacteria. Interestingly, the effect occurs in the presence of an insoluble material (activated carbon). Our findings suggest the involvement of multiple systems in electron transfer between species when consortia are supplemented with electron-active compounds. We conclude that a transition from acidogenesis (reducing power generation) to solventogenesis (reducing power demanding) can be shared between two bacterial species in a cooperative way governed at the gene expression level, which can be reinforced by the addition of external electron active compounds, paving the ground to potential gene editing (e.g. overexpression of *hyd* or *rnf* genes) of key activities.

## Data Availability

Raw RNA-sequencing data were submitted to the SRA (https://www.ncbi.nlm.nih.gov/) under project number PRJNA1345669.
